# Biodiversity and human well-being: an essential link for sustainable development

**DOI:** 10.1098/rspb.2016.2091

**Published:** 2016-12-14

**Authors:** Shahid Naeem, Robin Chazdon, J. Emmett Duffy, Case Prager, Boris Worm

**Affiliations:** 1 Department of Ecology, Evolution and Environmental Biology, Columbia University, New York, NY 10027, USA; 2 Department of Ecology and Evolutionary Biology, University of Connecticut, Storrs, CT 06269, USA; 3 Tennenbaum Marine Observatories Network, Smithsonian Institution, Washington, DC 20013, USA; 4 Department of Biology, Dalhousie University, Halifax, Nova Scotia, Canada B3H 4R2

**Keywords:** biodiversity, sustainable development, human well-being, ecosystem services

## Abstract

As society strives to transition towards more sustainable development pathways, it is important to properly conceptualize the link between biodiversity (i.e. genes, traits, species and other dimensions) and human well-being (HWB; i.e. health, wealth, security and other dimensions). Here, we explore how published conceptual frameworks consider the extent to which the biodiversity–HWB links are being integrated into public discourse and scientific research and the implications of our findings for sustainable development. We find that our understanding has gradually evolved from seeing the value of biodiversity as an external commodity that may influence HWB to biodiversity as fundamental to HWB. Analysis of the literature trends indicates increasing engagement with the terms *biodiversity*, *HWB* and *sustainable development* in the public, science and policy spheres, but largely as independent rather than linked terms. We suggest that a consensus framework for sustainable development should include biodiversity explicitly as a suite of internal variables that both influence and are influenced by HWB. Doing so will enhance clarity and help shape coherent research and policy priorities. We further suggest that the absence of this link in development can inadvertently lead to a ratcheting down of biodiversity by otherwise well-meaning policies. Such biotic impoverishment could lock HWB at minimum levels or lead to its decline and halt or reverse progress in achieving sustainable development.

## 1. Introduction

For several decades, world governments and policy bodies have been on a course of attempting to improve human well-being (HWB) through the stated intention of sustainable development, which includes improved education, health and environmental quality [1–9], although often to the exclusion of family planning and the demographic dividend (i.e. economic benefits associated with changes in age structure that occur when birth and death rates decline) in policy development [10,11]. Although biodiversity has long been considered integral to this sustainable development agenda [4,12–15], its relationship to HWB has not been systematically explored. As Seddon *et al*. [9] note, effective conservation, restoration and sustainable practice rest heavily on how clearly the science and policy spheres understand biodiversity's many values. Our motivation here is, through a systematic exploration of the current literature, examining its trends, its findings and its frameworks, to provide such clarity. Our focus, however, is specifically on biodiversity's values as they relate to improving HWB, the stipulated goal of sustainable development. Understanding the link between biodiversity and HWB is important as both parameters are undergoing considerable change.

*Biodiversity*, *ecosystem functioning*, *ecosystem services* and *human well-being* are widely used terms, though how they are defined, unfortunately, varies among sectors, sometimes generating confusion. Biodiversity is most commonly defined as the variability among living organisms from all sources including taxonomic, phylogenetic, and functional diversity and the ecological complexes of which they are part [16]. Though complex in definition, global syntheses focusing on species or other components have documented widespread loss of biodiversity [17–21]. Every ecosystem features key functions such as primary production and nutrient cycling, which give rise to ecosystem services that improve HWB, such as the provisioning of clean water, fertile soils, timber and capture fisheries [9,22–27].

*HWB*, like biodiversity [28], is a multidimensional construct that includes both subjective (e.g. how happy are you on a scale of 1–4) as well as objective measures (e.g. access to medical care) [29,30]. HWB has eluded any universal definition because of this multidimensionality [30]; it encompasses concepts of knowledge, friendship, self-expression, affiliation, bodily integrity, economic security, freedom, affection, wealth and leisure [31]. The Millennium Ecosystem Assessment (MA), for example, considered HWB to consist of five dimensions or elements: (i) basic material for a good life, (ii) security, (iii) health, (iv) good social relations, and (v) freedom of choice and action [2]. There are, however, many other subjective and objective variables that can be included [32,33]. In a review of HWB indices, for example, Smith *et al*. [32] identified 799 indicator variables, many of which relate to the MA's five HWB pillars.

Ecosystem functions and services are shaped by their biodiversity; it is intuitive that HWB and biodiversity should be linked. To date, two alternative (though not mutually exclusive) perspectives on the relationship between biodiversity and HWB have shaped public discourse and scientific research. One perspective emphasizes that human or economic development, in which natural, human, social and other capital stocks are marketed to produce flows of desired economic outputs, comes at the price of biodiversity loss. Such human development is often motivated by aspirations to improve HWB, but over recent history, this development has come at the expense of natural capital which has declined while other forms of capital, such as financial, social and built, have increased [34–36]. Indeed, processes directly and indirectly associated with declines in biological diversity have largely driven human colonization and development of civilizations on all continents since the Early- to Mid-Holocene about 5000–7000 yr ago [37–39]. These processes include conversion of natural habitats to agriculture [40–42], unsustainable exploitation of living resources [43], alteration of biogeochemical cycles [44], substitution of native and wild by exotic and domesticated species [45], freshwater appropriation and impoundment [46], human appropriation of primary production [47–49] and other human activities that generally lead to biodiversity loss [18,27,50–52]. More specifically, much of this biodiversity loss is linked directly to the explosive growth over recent decades in global trade in basic commodities, such as coffee, tea, sugar, textiles and fish [43,53]. This causal chain that links human development, biodiversity and HWB can be illustrated as follows:1.1
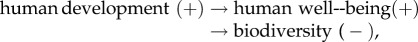
where parenthetical signs indicate increases (+) or decreases (−). This perspective has been concerned with biodiversity primarily as an external variable of unspecified, intrinsic value that is essentially affected as collateral damage during human development processes.

The second, newer perspective, emphasizes biodiversity as the foundation of a system that produces HWB via its positive effects on ecosystem function [9,54–61]. This can be illustrated as follows:1.2
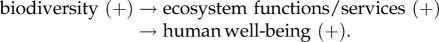
As we will show below, these two different perspectives lead to different frameworks which can generate confusion across sectors.

## 2. Biodiversity and human well-being linkages in existing conceptual frameworks

**Figure 1.**
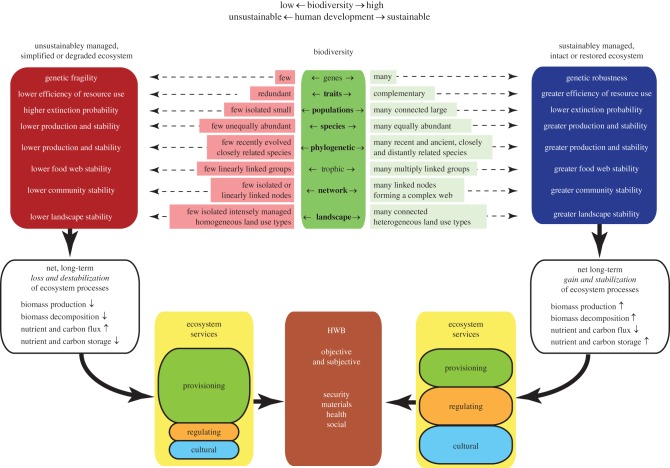
Linking economic development (sustainable or unsustainable), biodiversity, ecosystem functioning, ecosystem services and HWB. Biodiversity is illustrated centrally as a multidimensional construct (top, central green box) in which a biota varies in its diversity of genes, traits, species, and other dimensions. This biodiversity undergoes collective change (decline to the left, increase to the right), each dimension changing as described to the left (declining) or right (increasing) depending on management (unsustainable to the left, sustainable to the right) or other human interventions. The characteristics of these changes for each dimension are described in the boxes left and right of the biodiversity box. Change results in biodiversity-poor ecosystems (left, top) or biodiversity-rich ecosystems (right, top). Research has demonstrated, though results vary and knowledge gaps remain, that change in each dimension has different impacts on the magnitudes and stability of ecosystem functions which alter properties of ecosystems, as described in the top, left and top, right boxes. Development that leads to biodiversity-poor ecosystems results in a net loss and destabilization of ecosystem processes (left, white box) attributable to increases or decreases in ecosystem functions, only four of which are shown with up or down arrows to indicate increases or decreases. The converse occurs where development leads to biodiversity-rich ecosystems (right, white box). These contrasting changes in ecosystem functions lead to differences in ecosystem service delivery (boxes adjacent to bottom central box). Biodiversity-poor systems (e.g. monoculture production landscapes or collapsed open ocean fisheries) provide short-term, unstable increases in provisioning services with concomitant in regulating and cultural services (left). The converse occurs in systems managed to sustain biodiversity (right). HWB experiences change in its many components, here categorized as security, materials for a good life, all dimensions of mental and physical health, and good social relations in a stable and productive society. (Online version in colour.)

Because biodiversity, ecosystem functioning, ecosystem services and HWB are complex constructs, there are many linkages among them that makes the simultaneous consideration of all four constructs challenging. Figure 1, for example, considers just eight dimensions of biodiversity, four dimensions of ecosystem functioning, three dimensions of ecosystem services and four dimensions of HWB for two development pathways, which, in theory, consists of 768 (8 × 4 × 3 × 2) possible outcomes for a single change in biodiversity for a minimal set of dimensions for the four constructs. The frameworks we review seek ways to simplify these linkages.

Theoretical and empirical support are the strongest for the relationships among taxonomic, functional and to a limited extent, phylogenetic diversity and ecosystem function [9,28,56,57,61,62]. There are, however, considerable knowledge gaps on the links between biodiversity and ecosystem services [58,61,63]. Trade-offs and synergies among linkages are also poorly studied [55,64,65] (see, also, the literature survey, below). To the best of our knowledge, complete, quantitative studies that link biodiversity and HWB via ecosystem functions and services, have yet to be done, let alone tests of 768 possible outcomes of the minimal set of dimensions illustrated in figure 1.

**Figure 2.**
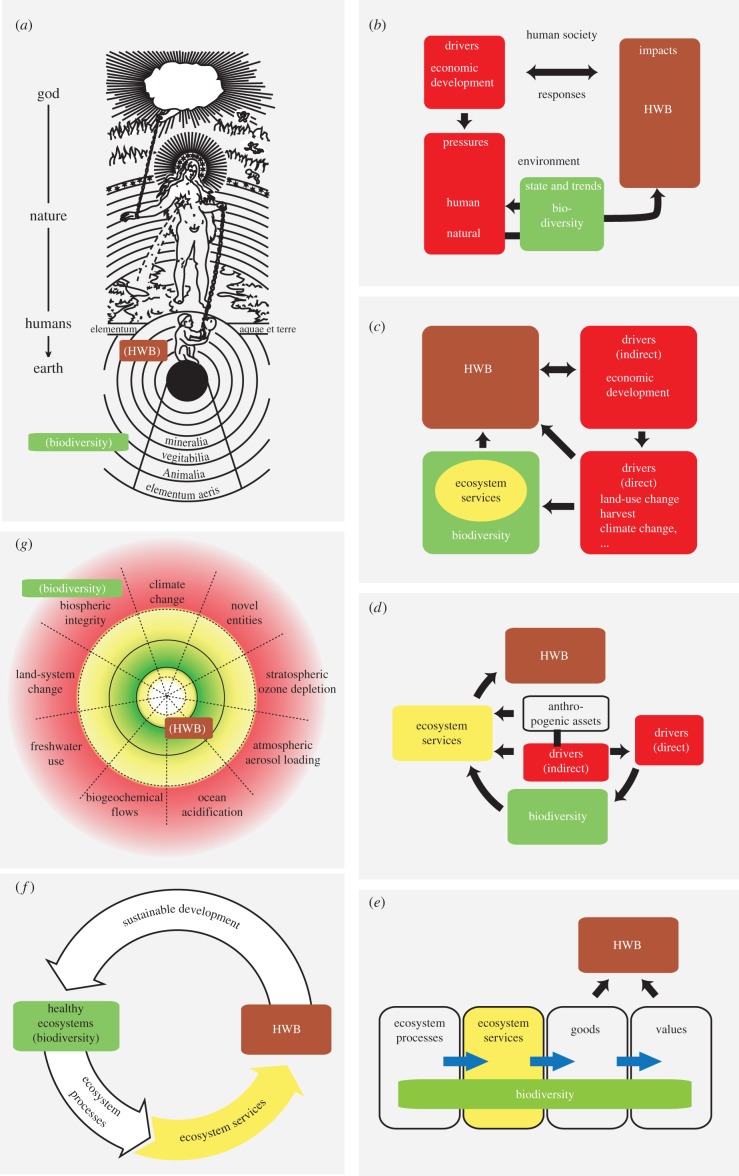
Conceptual frameworks. Shown are published frameworks that include biodiversity and HWB, explicitly or implicitly. (*a*) Reproduction of a fifteenth century Western understanding of the relationship between humans and nature (adapted from [66]). (*b*) The DPSIR framework (adapted from [67–69]). (*c*) The MA framework (adapted from [2]). (*d*) The IPBES framework (adapted from [70–72]). (*e*) Adapted from Mace *et al*. [63]. (*f*) Adapted from Rogers *et al*. [33] framework based on the construct of *healthy ecosystems*. (*g*) The safe planetary boundaries framework (adapted from [73]) currently uses the term ‘biotic integrity’ which implicitly refers to biodiversity, thus we have placed the term parenthetically in the framework. Likewise, we have placed HWB parenthetically in the framework taking the zones to implicitly reflect elements of HWB. Note that, to improve readability and reduce clutter, we have extracted only the core elements of each framework, focusing specifically on biodiversity (green boxes), human or economic development that is usually associated with drivers of change (red boxes), ecosystem services (yellow boxes) and HWB (brown boxes), leaving out complex features such as scale and tabulations of specific elements of biodiversity and HWB or examples of ecosystem services and ecosystem functions. Our purpose here is to show the multiplicity of ways in which biodiversity and HWB are related to one another by different frameworks, but these frameworks serve to illustrate many other relationships among a larger number of factors than we address here. See text for further explanation. (Online version in colour.)

In spite of the limited research on the linkages and outcomes illustrated in figure 1, several conceptual frameworks have nevertheless been developed (figure 2). In many frameworks, biodiversity is conceptualized as an external commodity influencing HWB, similar to clean air and water, or as a source of materials (e.g. pharmaceuticals, genetic resources for plant and animal breeding), recreation or other values [9]. Thus, HWB is not seen as something that emerges from biodiversity but as an amalgam of many factors of which biodiversity is just one that may provide positive physical and mental benefits [74] and be a source of resilience for ecosystem services important to HWB [75–78]. As such, HWB is an integrative construct similar to the total economic value or the economics of ecosystems and biodiversity [9].

On the other hand, more complex linkages between humanity and nature have long been recognized, such as seen in Robert Fludd's sixteenth century illustration (figure 2*a*). In his figure, humanity is seen as small and primitive but sitting atop a world structured by air, minerals and Earth's biota. Though his world was ruled by nature, seen as a nurturing force, nature in turn was ruled by God. Thus, HWB was largely seen as a matter of fate, the outcome of processes outside human influence, but our connections to nature were clear [66,79].

More contemporary scientific frameworks vary in their inclusion of biodiversity and HWB. Tapio & Willamo [67], for example, examined several environmental protection frameworks which emphasize human drivers or pressures that lead to environmental problems and adverse impacts on health, air, water and biodiversity. These impacts in turn elicit human responses designed to correct environmental problems. The driving forces–pressures–state–impacts–responses (DPSIR) framework [67–69] shown in figure 2*b* sees human development as the source of pressures that affect the environment that in turn affect HWB.

The MA [2], built on a decade of research into the functional importance of biodiversity [80] to provide a radically new perspective in positioning biodiversity as the foundation for ecosystem functioning and the services it provides (figure 2*c*). Here, biodiversity is illustrated as an all-encompassing factor that mediates ecosystem functions which influence HWB through the services biodiversity generates.

The Intergovernmental Platform on Biodiversity and Ecosystem Services (IPBES) [70–72] adopted a framework that incorporated elements of both the MA and the driver-impact-response framework. Biodiversity is combined with ecosystems and subsumed under the banner of ‘Nature’, whose sole output is ‘Nature's Benefits to People’ (figure 2*d*).

Mace *et al*. [63] addressed the confusion created by assessment frameworks, such as the MA, that saw biodiversity as both a driver of ecosystem functioning and an ecosystem service itself. Their solution was to embed biodiversity across a four-part framework (figure 2*e*), making it at once a regulator of ecosystem functions (processes), an ecosystem service and an ecosystem good.

Rogers *et al*. [33] embed biodiversity into the construct of *healthy ecosystems* (figure 2*f*). In this framework, biodiversity becomes a biotic factor, which coupled with abiotic factors collectively determines the flow of goods and services that influences HWB. HWB itself is treated in a separate framework and seen as an eight-dimensional construct, comprising both subjective and objective variables, one of which, *stable ecosystems*, includes biodiversity.

Finally, in a break from the box-and-arrow approach of most frameworks, the safe planetary boundaries framework [4,73,81] focuses on a limited set of key threats to the integrity of planetary processes and implicitly HWB (figure 2*g*). The original framework labelled one boundary ‘biodiversity loss’, but it is now labelled ‘biosphere integrity’, which is divided into functional and genetic diversity and offers the Biodiversity Intactness Index [82] and extinction rates (extinctions/million-species-years) as possible metrics for each, respectively.

This multiplicity of views illustrates widespread consensus on the links between biodiversity and HWB, but it also generates confusion.

## 3. Public and scientific uptake of the biodiversity–human well-being linkages

The link between biodiversity and HWB became a focus of public discourse and scientific research in the early 1990s following the Brundtland Report [1], and the United Nations (UN). Conference on Environment and Development held in Rio de Janeiro in 1992. The latter event also marked the launch of the UN Framework Convention on Climate Change and the UN Conventions on Biological Diversity and to Combat Desertification, all seen as landmarks in the rise of sustainable development as a societal paradigm. As described in the previous section, environmental frameworks always couple biodiversity and HWB, but not in consistent ways. Frameworks have also been developed separately by the research and policy sectors, and sometimes by both, which influences their accessibility and uptake by different sectors. Biodiversity is variously considered an externality, a driver or a diffuse variable, embedded in one or more parts of a framework. These deliberations beg the following question: given its ubiquity and variability in conceptual frameworks, how has the biodiversity–HWB link been taken up in public discourse and scientific research?

To address this question, we used text product databases as proxy measures of public discourse and scientific research. Using the LexisNexis Academic database, we quantified public discourse by tallying the number of text products, consisting of general news pieces (e.g. web-based publications, newspaper articles, law review articles, magazine articles) that referenced the following search terms: *biodiversity*, *human well-being* or *human wellbeing*, *sustainable development*, *biodiversity* and *human well-being* or *human wellbeing*, *biodiversity* and *sustainable development*, *human well-being* or *human wellbeing* and *sustainable development*, and *biodiversity* and *human well-being* or *human wellbeing* and *sustainable development*. As the number of text products returned for the single terms *biodiversity* and *sustainable development* from 1991 to 2014 exceeded the LexisNexis Academic limit on products retrieved (1000), we recorded the number of publications on the first day of each month and then summed and averaged those tallies, multiplying the end result by 365 to create an estimate of the number of products in a given year (we assumed there were no calendar biases such as spikes on the first day of the month or at the end of the year). For scientific research, we tallied peer-reviewed scientific publications in the ISI Web of Science database using the same term list, though LexisNexis required sampling to determine product output while ISI provided direct counts. We anchored our analyses to the base year 1985, a few years prior to the surge of interest generated by the Brundtland Report [1] and the 1992 Earth Summit. We note that focus on text products ignores other media that can be fairly important, such as film, video and other non-literary art forms.

**Figure 3.**
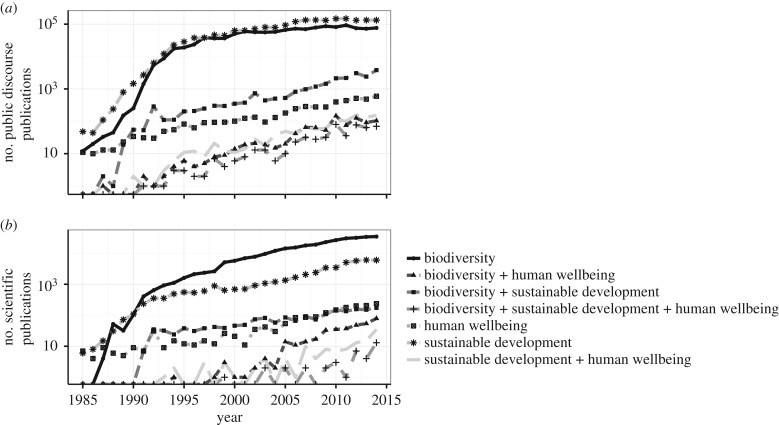
The literature survey. Shown are the log-transformed results of the literature searches for articles from 1985 to present mentioning the terms sustainable development, biodiversity and HWB, as well as their combinations. Two databases were used (*a*) LexisNexis, serving as a proxy for public discourse and (*b*) ISI Web of Science serving as a proxy for scientific research. See text for search protocol.

There are several striking features from the results of these text product analyses. First, public discourse shows an unprecedented, exponential rise in public engagement with the terms *sustainable development* and *biodiversity* that exceeded three orders of magnitude (figure 3, top), with the rise levelling by the mid-1990s around 10^5^ text products per year. This rise is possibly even more striking given that we do not include non-text-based products. Currently, an average of 350 text products published each day uses the single term *sustainable development* and around 200 products everyday uses *biodiversity*. By contrast, HWB as a single term rises more slowly with current daily output of roughly fewer than 3 per day.

Products that use any combination of terms have shown substantially lower engagement with most multi-term text products currently showing outputs of about 100 per year, the notable exception being products that use *biodiversity* + *sustainable development*. The lowest outputs are products using all three terms. Joint use of biodiversity and HWB has the rarest occurrence of two terms combined.

Scientific research shows a similar pattern to that of public discourse (figure 3, bottom). Not surprisingly, *biodiversity* occurs in scientific products more than any other term or set of terms as opposed to *sustainable development* in the public discourse products. That the magnitudes for the scientific literature outputs are substantially lower than text product outputs in public discourse is not surprising as it reflects the larger volume of text outputs in public discourse. Similar to public discourse, however, the number of papers that use combinations of the three terms are an order of magnitude lower than those that use the terms singly, whereas those using all three terms are rare. Joint use of biodiversity and HWB is roughly indistinguishable from the joint use of any other pair.

## 4. Discussion and synthesis

The fact that biodiversity and HWB are linked is well established. However, it appears from our brief surveys of frameworks and the literature that our present understanding of this link is variously posited as:

— biodiversity is a foundation of ecosystem processes/functions—its decline impairs the magnitude and stability of ecosystem functions that, in turn, adversely affects HWB. Indeed, Seddon *et al*. (this special feature [9]), propose *biodiversity services* as an alternative to *ecosystem processes* to emphasize biodiversity's foundational role;
— biodiversity is a product of ecosystem functioning—healthy ecosystems support more biodiversity;
— biodiversity is an environmental commodity, like clean air and water;
— biodiversity is intrinsically an ecosystem service—it is an ecosystem property we value in its own right; and
— biodiversity can be an element of HWB, like social cohesion, happiness and connections to nature, for some people and some cultures.

While these views are not mutually exclusive, they can differently influence public discourse (perceptions, actions and policy) as well as scientific research. Based on the literature trends, summing over the period between 1985 and 2014, the majority of our public discourse (99.2%) and research papers (99.3%) considers biodiversity, HWB and sustainable development as isolated constructs. The literature sources considering two of these terms were often an order of magnitude less common than those using terms singly, and those that considered all three terms together were rare (figure 3). There may be more overlap among these constructs than our methods detect depending on authors' selections of key words and terms, but given the high percentage (more than 90%), our findings are likely to be qualitatively accurate. It is also, perhaps, not surprising that individual terms may be used in isolation initially and collectively at a later time when integration occurs, which would explain the slower rise in multiple usages.

## 5. Synthesis

**Figure 4.**
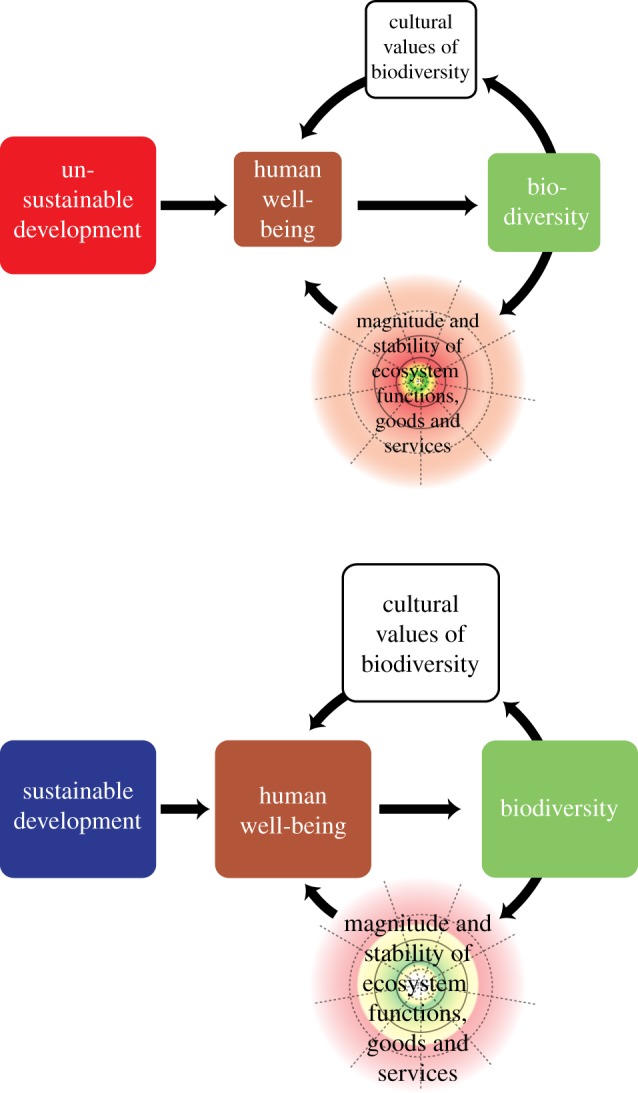
Simplified framework for sustainable development. This conceptual framework combines causalities from models 1 to 3 (see Introduction) and ideas captured in conceptual frameworks presented in figure 2 by establishing feedbacks from biodiversity to HWB. These feedbacks can be direct, where people appreciate biodiversity for its tangible (e.g. birding or snorkelling) or less tangible (e.g. inspirational or aesthetic) cultural values, or indirect, via the stable provisioning of ecosystem goods and services at magnitudes that support HWB. Sustainable development strives to optimize increases in HWB with the maintenance and restoration of biodiversity, establishing a positive feedback. Note that unsustainable development leads to a reduction in biodiversity, benefits derived from cultural values of biodiversity and HWB illustrated as smaller boxes than the bottom illustration for sustainable development. Note also that the safe space, illustrated as the yellow and green regions in the circular areas under the magnitude and stability of ecosystem functions, goods and services, also shrinks under unsustainable development. (Online version in colour.)

Our brief review reveals a range of perspectives on the linkage between biodiversity and HWB, and calls for a more coherent and unified framework. In figure 4, we present a unified framework that includes both the effects of human development on biodiversity and well-being and feedbacks from biodiversity to HWB. From this unified framework, it becomes clear that development will be sustainable when it strives to minimize harmful feedbacks, and ideally turns them into beneficial feedbacks by restoring biodiversity where it has been degraded. Rather than allowing natural capital (e.g. fossil fuels, soil, non-renewable minerals, old-growth forest, bushmeat and fish stocks) to be spent down by unsustainable development practices, we can insure long-term environmental sustainability by developing strategies for sustainable agriculture, forestry, animal husbandry and fisheries. These strategies, along with monitoring and tracking, should be adaptive and integrated. The SDGs, for example, could better meet their 2030 targets by greater integration of biodiversity into the 17 goals rather than separating them into 15 goals concerning HWB and only two concerning biodiversity (see also Seddon *et al*. [9]).

In seeking to maximize gains in HWB, distinguishing between achieving a robust versus fragile state of HWB is important (e.g. ‘thick’ and ‘thin’ well-being as described by [83]). A fragile state of HWB is one focused primarily on securing the immediate survival needs of people affected by poor nutrition, clean water shortage and poverty (akin to the physiological needs in Maslow's hierarchy of human needs [84]), while a robust state of HWB is one that achieves minimums, is resistant to shocks, and has the potential to rise above minimums. Whether one subscribes to an optimistic or pessimistic view for likely future trends in HWB, current levels of poverty, hunger and water scarcity call for urgent actions to improve and reduce inequality in HWB as quickly as possible. Focusing on securing minimum levels of HWB, however, may mean securing just enough biodiversity to insure minimum levels in the psychological, physiological, social, aesthetic, heath, material benefits and ecological resiliency biodiversity provides (i.e. the MA's 4 pillars of HWB [85]). In the interim, however, if biodiversity loss is prevalent and irreversible, then moving to a more robust state and higher levels of HWB could be untenable.

This scenario in which urgency necessitates immediate pursuit of minimal HWB that allows for further losses in biodiversity that, in turn, leads to further declines in HWB, yields a dynamic in which biodiversity is steadily ratcheted downward. The possibly that a threshold exists and is crossed in which ecosystem functions and services change dramatically becomes increasingly likely in the face of this biodiversity–HWB ratchet. Given the relationship between biodiversity and poverty alleviation (Roe *et al*. this special feature [86]), this ratcheting down of biodiversity could co-ratchet poverty upward.

An alternative approach is to focus on achieving a robust state of HWB, one that leads to the preservation or retention of biodiversity. This alternative approach follows the strategies of prudent businesses that employ the precautionary principle; invest some part of their profits in protecting their capital through insurance, security, and research and development. In the same way that a prudent business is pre-adapted to future change in markets, an ecosystem rich in diversity is one that is pre-adapted to future environmental change. To maintain the ecosystem services on which HWB depends, we need to develop policy that requires investment (and/or conservation) to protect and value the forms of natural capital that generate those services.

The biodiversity–HWB ratchet is avoidable through better understanding and communicating the biodiversity–HWB link. The two frameworks we present in figure 4, for example, include safe planetary boundaries that draw attention to pursuing strategies for sustainable development that emphasize maintaining levels of biodiversity that are not minimally sufficient to get the job done, but sufficient to ensure robust Earth-system function. Building public discourse and research on the biodiversity–HWB link, promoting robust HWB and addressing the feedbacks, benefits and trade-offs associated with biodiversity-based HWB (e.g. [87]) are all important steps towards sustainable development.

## 6. Conclusion

Although both HWB and biodiversity are multidimensional constructs that can be difficult to define and quantify, their linkage must be a central feature of any conceptual framework that informs sustainable development. Biodiversity, however, is often seen as a diffuse agent, and often its importance is implied rather than explicitly incorporated. In this review, we find that biodiversity, HWB and sustainable development are typically treated in isolation and their linkages are neglected. A more robust framework would include both the effects of development on HWB and biodiversity, as well as feedbacks (figure 4). While there may appear to be some circularity in advocating biodiversity conservation to improve HWB and improving HWB to conserve biodiversity, the story is more complex. First, the biodiversity–HWB ratchet described above points to the threat of diversity levels becoming low enough to cross threshold levels and trigger potentially irreversible and detrimental changes in ecosystem processes, services and HWB. Second, there are ethical arguments against human transformations at scales that jeopardize earth-system functioning. Finally, human appropriation of natural resources needs to be controlled in order to secure biodiversity levels sufficient to insure robust levels of HWB that are well above minimums. Improved conceptual frameworks, and the discourse and research they instigate can help shape a sustainable development agenda that goes beyond securing immediate survival needs to create a society that values the restoration of biodiversity as both a base condition and a product of improved HWB.

## References

[1] WCED WC.OE.a.D. 1987 Our common future, report of the World Commission on Environment and Development, p. 400 Oxford, UK: Oxford University Press.

[2] MEA. 2005 Living beyond our means: natural assets and human well-being: statement from the Board, p. 24 Millennium Assessment.

[3] SDSN. 2013 An action agenda for sustainable development (ed. Network LC.o.t.SD.S.) New York, NY: United Nations.

[4] Griggs D et al. 2013 Policy: sustainable development goals for people and planet. Nature 495, 305–307. (10.1038/495305a) 23518546

[5] Murphy DA 2010 Sustainable development: from Brundtland to Rio 2012, p. 26 New York, NY: United Nations.

[6] Pachauri RK 2007 Sustainable well-being. Science 315, 913 (10.1126/science.1140975) 17303720

[7] Folke C, Carpenter S, Elmqvist T, Gunderson L, Holling CS, Walker B 2002 Resilience and sustainable development: building adaptive capacity in a world of transformations. AMBIO: J. Hum. Environ. 31, 437–440. (10.1579/0044-7447-31.5.437) 12374053

[8] Chivian D et al. 2008 Environmental genomics reveals a single-species ecosystem deep within earth. Science 322, 275–278. (10.1126/science.1155495) 18845759

[9] Seddon N, Mace GM, Pigot AL, Naeem S, Mouillot D, Tobias JA, Walpole M, Vause J 2016 Biodiversity in the Anthropocene: prospects and policy. Proc. R. Soc. B 282, 20151602 (10.1098/rspb.2015.1602) PMC5204156 27928040

[10] Lutz W 2014 A population policy rationale for the twenty-first century. Popul. Dev. Rev. 40, 527–544. (10.1111/j.1728-4457.2014.00696.x)

[11] Petruney T, Wilson LC, Stanback J, Cates W Jr 2014 Family planning and the post-2015 development agenda. Bull. World Health Organ. 92, 548 (10.2471/BLT.14.142893) 25177066 PMC4147409

[12] National Research Council B.o.SD, Policy Division. 2000 Our common journey: a transition toward sustainability, 384 Washington, DC: National Academy Press.

[13] UN. 2015 Millennium development goals report 2015. New York, NY: United Nations.

[14] Sachs JD et al. 2009 Biodiversity conservation and the millennium development goals. Science 325, 1502–1503. (10.1126/science.1175035) 19762629

[15] Adams WM, Aveling R, Brockington D, Dickson B, Elliott J, Hutton J, Roe D, Vira B, Wolmer W 2004 Biodiversity conservation and the eradication of poverty. Science 306, 1146–1149. (10.1126/science.1097920) 15539593

[16] CBD S.o.t.C.o.BD. 2005 Handbook of the convention on biological diversity including its cartegana protocol on biosafety, 3rd edn, p. 1493 Montreal, Canada: Secretariat of the Convention on BIological Diversity.

[17] Butchart SHM et al. 2010 Global biodiversity: indicators of recent declines. Science 328, 1164–1168. (10.1126/science.1187512) 20430971

[18] Dirzo R, Young HS, Galetti M, Ceballos G, Isaac NJB, Collen B 2014 Defaunation in the Anthropocene. Science 345, 401–406. (10.1126/science.1251817) 25061202

[19] Schipper J et al. 2008 The status of the world's land and marine mammals: diversity, threat, and knowledge. Science 322, 225–230. (10.1126/science.1165115) 18845749

[20] Davidson AD, Boyer AG, Kim H, Pompa-Mansilla S, Hamilton MJ, Costa DP, Ceballos G, Brown JH 2012 Drivers and hotspots of extinction risk in marine mammals. Proc. Natl Acad. Sci. USA 109, 3395–3400. (10.1073/pnas.1121469109) 22308490 PMC3295301

[21] Worm B, Branch TA 2012 The future of fish. Trends Ecol. Evol. 27, 594–599. (10.1016/j.tree.2012.07.005) 22877983

[22] Ehrlich PR, Mooney HA 1983 Extinction, substitution, and ecosystem services. BioScience 33, 248–253. (10.2307/1309037)

[23] Daily GC et al. 1997 Ecosystem services: benefits supplied to human societies by natural ecosystems. Issues Ecol. 2, 1–18.

[24] Daily GC, Matson PA 2008 Ecosystem services: from theory to implementation. Proc. Natl Acad. Sci. USA 105, 9455–9456. (10.1073/pnas.0804960105) 18621697 PMC2474530

[25] Palumbi SR et al. 2008 Managing for ocean biodiversity to sustain marine ecosystem services. Front. Ecol. Environ. 7, 204–211. (10.1890/070135)

[26] Cardinale BJ, Srivastava DS, Emmett Duffy J, Wright JP, Downing AL, Sankaran M, Jouseau C 2006 Effects of biodiversity on the functioning of trophic groups and ecosystems. Nature 443, 989–992. (10.1038/nature05202) 17066035

[27] Worm B et al. 2006 Impacts of biodiversity loss on ocean ecosystem services. Science 314, 787–790. (10.1126/science.1132294) 17082450

[28] Naeem S et al. 2016 Biodiversity as a multidimensional construct: a review, framework and case study of herbivory's impact on plant biodiversity. Proc. R. Soc. B 282, 20153005 (10.1098/rspb.2015.3005) PMC5204135 27928041

[29] Oswald AJ, Wu S 2010 Objective confirmation of subjective measures of human well-being: evidence from the USA. Science 327, 576–579. (10.1126/science.1180606) 20019249

[30] Agarwala M, Atkinson G, Fry BP, Homewood K, Mourato S, Rowcliffe JM, Wallace G, Milner-Gulland EJ 2014 Assessing the relationship between human well-being and ecosystem services: a review of frameworks. Conserv. Soc. 12, 437–449. (10.4103/0972-4923.155592)

[31] McGillivray M 2007 Human well-being: concept and measurement. Basingstoke, UK: Palgrave Macmillan.

[32] Smith LM, Case JL, Smith HM, Harwell LC, Summers JK 2013 Relating ecoystem services to domains of human well-being: foundation for a US. index. Ecol. Indic. 28, 79–90. (10.1016/j.ecolind.2012.02.032)

[33] Rogers DS, Duraiappah AK, Antons DC, Munoz P, Bai X, Fragkias M, Gutscher H 2012 A vision for human well-being: transition to social sustainability. Curr. Opin. Environ. Sustain. 4, 61–73. (10.1016/j.cosust.2012.01.013)

[34] Ehrlich PR, Kareiva PM, Daily GC 2012 Securing natural capital and expanding equity to rescale civilization. Nature 486, 68–73. (10.1038/nature11157) 22678281

[35] Pretty J, Ward H 2001 Social capital and the environment. World Dev. 29, 209–227. (10.1016/S0305-750X(00)00098-X)

[36] Costanza R, Daly HE 1992 Natural capital and sustainable development. Conserv. Biol. 6, 37–46. (10.1046/j.1523-1739.1992.610037.x)

[37] Grayson D 2001 The Archaeological record of human impacts on animal populations. J. World Prehistory 15, 1–68. (10.1023/A:1011165119141)

[38] Steadman DW 1995 Prehistoric extinctions of Pacific island birds: biodiversity meets zooarchaeology. Science 267, 1123–1131. (10.1126/science.267.5201.1123) 17789194

[39] Burney DA, Flannery TF 2005 Fifty millennia of catastrophic extinctions after human contact. Trends Ecol. Evol. 20, 395–401. (10.1016/j.tree.2005.04.022) 16701402

[40] Ramankutty N, Evan AT, Monfreda C, Foley JA 2008 Farming the planet: 1. Geographic distribution of global agricultural lands in the year 2000. Glob. Biogeochem. Cycles 22, GB1003. (10.1029/2007gb002952)

[41] Foley JA et al. 2005 Global consequences of land use. Science 309, 570–574. (10.1126/science.1111772) 16040698

[42] Newbold T et al. 2015 Global effects of land use on local terrestrial biodiversity. Nature 520, 45–50. (10.1038/nature14324) 25832402

[43] Lotze HK et al. 2006 Depletion, degradation, and recovery potential of estuaries and coastal seas. Science 312, 1806–1809. (10.1126/science.1128035) 16794081

[44] Vitousek PM, Aber J, Howarth RW, Likens GE, Matson PA, Schindler DW, Schlesinger WH, Tilman GD 1997 Human alteration of the global nitrogen cycle: causes and consequences. In Issues in ecology, pp. 1–15. Washington, DC: Ecological Society of America.

[45] Diamond J 2002 Evolution, consequences and future of plant and animal domestication. Nature 418, 700–707. (10.1038/nature01019) 12167878

[46] Postel SL, Daily GC, Ehrlich PR 1996 Human appropriation of renewable fresh water. Science 271, 785–788. (10.1126/science.271.5250.785)

[47] Vitousek P, Ehrlich P, Ehrlich A, Matson PA 1986 Human appropriation of the products of photosynthesis. BioScience 36, 368–373. (10.2307/1310258)

[48] Haberl H, Erb KH, Krausmann F, Gaube V, Bondeau A, Plutzar C, Gingrich S, Lucht W, Fischer-Kowalski M 2007 Quantifying and mapping the human appropriation of net primary production in earth's terrestrial ecosystems. Proc. Natl Acad. Sci. USA 104, 12 942–12 947. (10.1073/pnas.0704243104) PMC1911196 17616580

[49] Krausmann F, Erb K-H, Gingrich S, Haberl H, Bondeau A, Gaube V, Lauk C, Plutzar C, Searchinger TD 2013 Global human appropriation of net primary production doubled in the 20th century. Proc. Natl Acad. Sci. USA 110, 10 324–10 329. (10.1073/pnas.1211349110) PMC3690849 23733940

[50] Sala E, Knowlton N 2006 Global marine biodiversity trends. Annu. Rev. Environ. Resour. 31, 93–122. (10.1146/annurev.energy.31.020105.100235)

[51] Ceballos G, Ehrlich PR, Barnosky AD, García A, Pringle RM, Palmer TM 2015 Accelerated modern human–induced species losses: entering the sixth mass extinction. Sci. Adv. 1, e1400253 (10.1126/sciadv.1400253) 26601195 PMC4640606

[52] Gren I-M, Campos M, Gustafsson L 2015 Economic development, institutions, and biodiversity loss at the global scale. Reg. Environ. Change 16, 1–13. (10.1007/s10113-015-0754-9)

[53] Lenzen M, Moran D, Kanemoto K, Foran B, Lobefaro L, Geschke A 2012 International trade drives biodiversity threats in developing nations. Nature 486, 109–112. (10.1038/nature11145) 22678290

[54] Millennium Ecosystem Assessment. 2005 Ecosystems and human well-being: synthesis. Washington, DC: Island Press.

[55] Bennett EM et al. 2015 Linking biodiversity, ecosystem services, and human well-being: three challenges for designing research for sustainability. Curr. Opin. Environ. Sustain. 14, 76–85. (10.1016/j.cosust.2015.03.007)

[56] Naeem S, Duffy JE, Zavaleta E 2012 The functions of biological diversity in an age of extinction. Science 336, 1401–1406. (10.1126/science.1215855) 22700920

[57] Tilman D, Isbell F, Cowles JM 2014 Biodiversity and ecosystem functioning. Annu. Rev. Ecol. Evol. Syst. 45, 471–493. (10.1146/annurev-ecolsys-120213-091917)

[58] Harrison PA et al. 2014 Linkages between biodiversity attributes and ecosystem services: a systematic review. Ecosyst. Serv. 9, 191–203. (10.1016/j.ecoser.2014.05.006)

[59] Aicher C, Wittmer H, Schröter-Schlaack C, Rode J, Hansjürgens B 2015 The multifaceted contribution of biodiversity to human well-being: lessons from The Economics of Ecosystems and Biodiversity (TEEB) initiative. In Biodiversity in the green economy (Routledge Studies in ecological economics) (eds Gasparatos A, Willis KJ), pp. 383–401. Abingdon, UK: Routeledge.

[60] Chazdon RL 2008 Beyond deforestation: restoring forests and ecosystem services on degraded lands. Science 320, 1458–1460. (10.1126/science.1155365) 18556551

[61] Cardinale BJ et al. 2012 Biodiversity loss and its impact on humanity. Nature 486, 59–67. (10.1038/nature11148) 22678280

[62] Turnbull

[63] Mace GM, Norris K, Fitter AH 2012 Biodiversity and ecosystem services: a multilayered relationship. Trends Ecol. Evol. 27, 19–26. (10.1016/j.tree.2011.08.006) 21943703

[64] Summers JK, Smith LM, Case JL, Linthurst RA 2012 A review of the elements of human well-being with an emphasis on the contribution of ecosystem services. Ambio 41, 327–340. (10.1007/s13280-012-0256-7) 22581385 PMC3393065

[65] Howe C, Suich H, Vira B, Mace GM 2014 Creating win-wins from trade-offs? Ecosystem services for human well-being: a meta-analysis of ecosystem service trade-offs and synergies in the real world. Glob. Environ. Change 28, 263–275. (10.1016/j.gloenvcha.2014.07.005)

[66] Naeem S 2002 Ecosystem consequences of biodiversity loss: the evolution of a paradigm. Ecology 83, 1537–1552. (10.1890/0012-9658(2002)083%5B1537:ECOBLT%5D2.0.CO;2)

[67] Tapio P, Willamo R 2008 Developing interdisciplinary environmental frameworks. AMBIO: A J. Hum. Environ. 37, 125–133. (10.1579/0044-7447(2008)37%5B125:DIEF%5D2.0.CO;2) 18488556

[68] Maxim L, Spangenberg JH, O'Connor M 2009 An analysis of risks for biodiversity under the DPSIR framework. Ecol. Econ. 69, 12–23. (10.1016/j.ecolecon.2009.03.017)

[69] UNEP. 2012 Global Environment Outlook: GEO5: environment for the future we want, p. 528.

[70] Díaz S et al. 2015 The IPBES Conceptual Framework — connecting nature and people. Curr. Opin. Environ. Sustain. 14, 1–16. (10.1016/j.cosust.2014.11.002)

[71] Larigauderie A, Mooney HA 2010 The Intergovernmental science-policy Platform on Biodiversity and Ecosystem Services: moving a step closer to an IPCC-like mechanism for biodiversity. Curr. Opin. Environ. Sustain. 2, 9–14. (10.1016/j.cosust.2010.02.006)

[72] Larigauderie A et al. 2012 Biodiversity and ecosystem services science for a sustainable planet: the DIVERSITAS vision for 2012–20. Curr. Opin. Environ. Sustain. 4, 101–105. (10.1016/j.cosust.2012.01.007) 25104977 PMC4121961

[73] Steffen W et al. 2015 Planetary boundaries: Guiding human development on a changing planet. Science 1259855 (10.1126/science.1259855) 25592418

[74] Sandifer PA, Sutton-Grier AE, Ward BP 2015 Exploring connections among nature, biodiversity, ecosystem services, and human health and well-being: opportunities to enhance health and biodiversity conservation. Ecosyst. Serv. 12, 1–15. (10.1016/j.ecoser.2014.12.007)

[75] Elmqvist T, Folke C, Nyström M, Peterson G, Bengtsson J, Walker B, Jon N 2003 Response diversity, ecosystem change, and resilience. Front. Ecol. Environ. 1, 488–494. (10.1890/1540-9295(2003)001%5B0488:RDECAR%5D2.0.CO;2)

[76] Folke C, Carpenter S, Walker B, Scheffer M, Elmqvist T, Gunderson L, Holling CS 2004 Regime shifts, resilience, and biodiversity in ecosystem management. Annu. Rev. Ecol. Evol. Syst. 35, 557–581. (10.1146/annurev.ecolsys.35.021103.105711)

[77] Thompson I 2011 Biodiversity, ecosystem thresholds, resilience and forest degradation. Unasylva, 25–30.

[78] Díaz S, Fargione J, Chapin FS III, Tilman D 2006 Biodiversity loss threatens human well-being. PLoS Biol. 4, e277 (10.1371/journal.pbio.0040277) 16895442 PMC1543691

[79] Naeem S 2013 Ecosystem services: is a planet serving one species likely to function? In Linking ecology and ethics for a changing world: values, philosophy, and action (eds Rozzi R, Pickett STA), pp. 303–321. Berlin, Germany: Springer.

[80] Schulze ED, Mooney HA 1993 Biodiversity and ecosystem function. New York, NY: Springer.

[81] Rockstrom J et al. 2009 A safe operating space for humanity. Nature 461, 472–475. (10.1038/461472a) 19779433

[82] Scholes RJ, Biggs R 2005 A biodiversity intactness index. Nature 434, 45–49. (10.1038/nature03289) 15744293

[83] Moore A 1994 Well-being: a philosophical basis for health services. Health Care Anal. 2, 207–216. (10.1007/BF02251021) 10137627

[84] Maslow AH 1943 A theory of human motivation. Psychiol. Rev. 50, 370–396. (10.1037/h0054346)

[85] MEA. 2005 Ecosystems and human well-being. Washington, DC: Island Press.

[86] Roe D, Sunderland TCH, Reed J, van Vianen J, Seddon N 2016 The good, the bad and the ugly: the value of biodiversity for poverty alleviation. Proc. R. Soc. B 283.

[87] McShane TO et al. 2011 Hard choices: making trade-offs between biodiversity conservation and human well-being. Biol. Conserv. 144, 966–972. (10.1016/j.biocon.2010.04.038)

